# A functional evaluation of feeding in the surgeonfish *Ctenochaetus striatus*: the role of soft tissues

**DOI:** 10.1098/rsos.171111

**Published:** 2018-01-31

**Authors:** Sterling B. Tebbett, Christopher H. R. Goatley, Víctor Huertas, Michalis Mihalitsis, David R. Bellwood

**Affiliations:** 1 ARC Centre of Excellence for Coral Reef Studies, James Cook University, Townsville, Queensland 4811, Australia; 2 College of Science and Engineering, James Cook University, Townsville, Queensland 4811, Australia; 3 Function, Evolution and Anatomy Research (FEAR) Lab and Palaeoscience Research Centre, School of Environmental and Rural Science, University of New England, Armidale, New South Wales 2351, Australia

**Keywords:** coral reef fish, detritivory, morphology, Great Barrier Reef, sediment, Acanthuridae

## Abstract

*Ctenochaetus striatus* is one of the most abundant surgeonfishes on Indo-Pacific coral reefs, yet the functional role and feeding ecology of this species remain unclear. This species is reported to possess a rigid structure in its palate that is used for scraping, but some authors have reported that this element is comprised of soft tissue. To resolve the nature and role of this structure in the feeding ecology of *C. striatus* we examined evidence from anatomical observations, scanning electron microscopy, histology, X-ray micro-computed tomography scanning, high-speed video and field observations. We found that *C. striatus* from the Great Barrier Reef possess a retention plate (RP) on their palates immediately posterior to the premaxillary teeth which is soft, covered in a thin veneer of keratin with a papillate surface. This RP appears to be used during feeding, but does not appear to be responsible for the removal of material, which is achieved primarily by a fast closure of the lower jaw. We infer that the RP acts primarily as a ‘dustpan’, in a ‘dustpan and brush’ feeding mechanism, to facilitate the collection of particulate material from algal turfs.

## 1. Introduction

Coral reef fishes exhibit a striking diversity of morphologies and behaviours which allow them to contribute to a wide range of ecosystem processes [1,2]. Understanding how fishes deliver specific functions can shed new light on their contribution to these processes. In many cases, a few species contribute disproportionately to particular functions because they are widely distributed, highly abundant, or because of a high degree of ecological specialization and limited functional redundancy [3,4]. This is particularly applicable in the case of the surgeonfish *Ctenochaetus striatus*. This fish occurs on coral reefs across 190° of longitude from the Red Sea to the central Pacific [5,6]. Throughout this range, it is often the most abundant surgeonfish [7–10]. Despite ongoing discussion in the literature [11], it is becoming increasingly clear that *C. striatus* plays a critical role in a number of key ecosystem processes on coral reefs, including sediment dynamics [12,13] and detritivory [11,14,15].

On coral reefs *C. striatus* feeds on components of the epilithic algal matrix (EAM), selectively brushing detritus and associated particulates from algal turfs [11]; the term detritus is used in a broad sense to describe amorphous organic particulate material that is likely to contain living material such as bacteria, microalgae, micro-invertebrates and fungi [16–18]. The brushing action is achieved through the use of highly modified comb-like teeth and jaw modifications which allow *C. striatus* to expand their jaws to nearly 180° [19–22]. While most authors agree on the unique structure and function of the comb-like teeth of *C. striatus*, there remains some disagreement over the nature and function of a structure located on the palate of *C. striatus*.

Krone *et al*. [23] identified a distinct structure on the upper palate of *C. striatus* as ‘rigid’ and containing ‘numerous single hard knobs' and, along with others, suggested that this structure is used for bioerosion [13,23,24]. Fishelson & Delarea [25] termed the structure in surgeonfishes located posteriorly to the teeth but anteriorly to the buccal valve, the ‘retention plate’ (RP; the term maintained herein). Fishelson & Delarea [25] noted the enlarged nature of the RP in *C. striatus* compared to other surgeonfishes and the pronounced cornified papillae on the RP, based on scanning electron microscopy (SEM) and transmission electron microscopy, but they made few ecological inferences. Questions about the nature of the RP and its use in bioerosion were also raised by Bonaldo *et al*. [26] who suggested that it is largely comprised of soft tissue. These contradicting studies all highlight that there is something unusual about the RP in *C. striatus*, but the exact functional role of this RP during feeding has not been determined. While a substantial body of literature exists on the palatal structures in fishes, including the pharyngeal organ [27,28] and buccal valves [29–31], and on the keratinized nature of palatal tissues (e.g. parakeratinized or orthokeratinized) in other vertebrates [32–34], our understanding of the nature and role of palatal structures in coral reef fishes is limited. However, the soft anatomy of the jaws can play an important role in reef fish feeding [35] and Clements *et al*. [17] have recently suggested the need for a reassessment of the soft trophic anatomy of herbivorous and detritivorous reef fishes.

It has also been suggested that *C. striatus* from the Red Sea and Andaman Sea employ a secondary feeding mode that involves ‘chafing’ the substratum using the RP [23,24,36]. In this feeding mode, the fish use ‘energetic grasping bites with contact pressure being generated by a shaking of the whole body’ [23], potentially allowing the RP to bioerode the substratum. This feeding behaviour differs markedly from the primary feeding mode which generally involves a quick upwards closure of the lower jaws that exerts little pressure on the substratum [21]. The frequency with which the chafing feeding mode is used would indicate the likelihood of bioerosion by *C. striatus*, yet it has not been quantified in the field nor has it been reported from the Great Barrier Reef (GBR). In this study, we aimed to determine the functional role of the RP and any chafing behaviour used during feeding by *C. striatus*. To accomplish this we used a range of data collected from field observations, dissections, SEM, X-ray micro-computed tomography (micro-CT) scanning, histological sections and high-speed video recordings.

## 2. Material and methods

### 2.1. Overview

**Figure 1.**
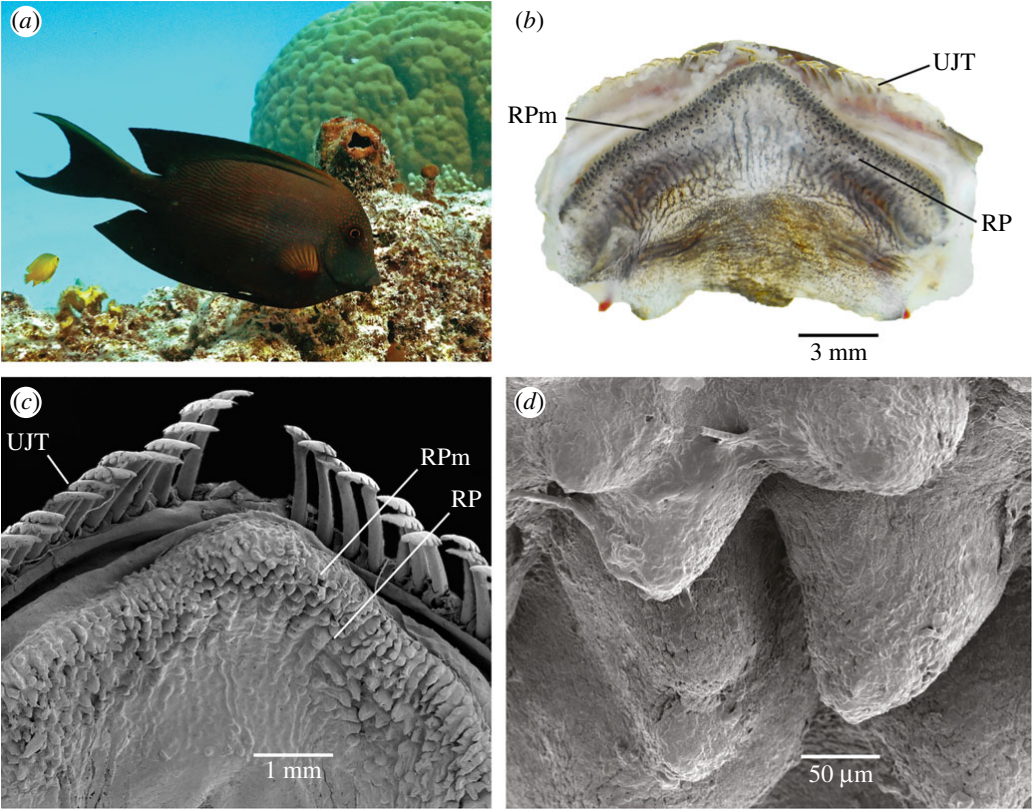
(*a*) The surgeonfish *Ctenochaetus striatus*, (*b*) the soft RP structure on the upper palate of *C. striatus* (UJT, upper jaw teeth; RP, retention plate; RPm, retention plate margin), (*c*) scanning electron micrograph (SEM) of the RP of *C. striatus*, (*d*) close up SEM of papillae covered surface of the RP.

All *C. striatus* (figure 1*a*) used for morphological examinations and behavioural recording in aquaria were adults between 145 and 235 mm total length (TL) (see electronic supplementary material, table S1, for full details). All specimens were collected from the GBR, mainly from around Lizard Island in the northern GBR. Specimens used for morphological examinations were euthanized with clove oil, then immediately immersed in an ice–water slurry.

### 2.2. Morphological examinations

#### 2.2.1. Anatomical observations

We performed tactile examinations of 12 specimens to determine the resistance of the RP to compression. We also performed visual examinations of the RP using a dissecting microscope (Olympus SZ61).

#### 2.2.2. Scanning electron microscopy

The upper palate, premaxilla and maxilla were removed from two *C. striatus* specimens (189 and 200 mm TL) and stored in a −80°C freezer (Snijders Labs EvoSafe VF-475-86) overnight. The samples were then freeze dried (using an Alpha 1–2 LDplus freeze dryer) at −55°C in vacuum, mounted on stubs, sputter coated with gold in a JEOL JUC-5000 magnetron sputtering device, and examined in a scanning electron microscope (JEOL JSM-5410LV).

#### 2.2.3. Histology

The anterior portion of the head was removed from two specimens (181 and 235 mm TL) and fixed in Bouin's fixative [37] for 48 h. Samples were then rinsed, stored in 70% ethanol, and decalcified for 48 h in Gooding & Stewart's decalcifying fluid [38]. Each sample was divided along the midline into left and right sections and dehydrated through a graded ethanol series (70% to absolute), cleared in xylene and paraffin-embedded. Sagittal sections (5 µm) were obtained using a MicroTec CUT4060 rotary microtome, mounted on glass slides, and stained. We used the Alcian blue-PAS (AB-PAS) stain [39] to test for the presence of reactive mucopolysaccharides, which would indicate mucus secretion in the RP. This stain has proved to be a reliable technique for detecting glycoproteins in a broad range of animals [39], including fishes [40]. We also used the Ayoub–Shklar staining technique to evaluate the occurrence of keratinization in the RP [41]. Photomicrographs were taken using an Olympus SZ40 stereo microscope equipped with an SZ-CTV adapter and an Olympus DP21 digital camera. The thickness of the keratinized layer was measured at 10 haphazardly selected points along the RP to estimate its average thickness (using Adobe Illustrator CC 2017).

#### 2.2.4. Three-dimensional modelling and density estimates

A three-dimensional (3D) model of *C. striatus* was generated from micro-CT data. A single specimen (170 mm TL), preserved in 70% ethanol, was scanned using a GE phoenix v|tome|x s industrial micro-CT scanner, with a slice increment of 63 µm. Image slices were constructed into a 3D surface mesh and the skull segmented from the body using Materialise Mimics Innovation Suite v.19.0 (Materialise NV, Leuven, Belgium)*.* The density of the RP was estimated by extracting greyscale data along four transects (higher values representing whiter/denser material), which ran from the midpoint of the right premaxilla towards the midpoint of the left premaxilla through the area of the RP.

### 2.3. Feeding observations

#### 2.3.1. Feeding observations in aquaria

To observe the use of the RP in detail, the feeding behaviour of two *C. striatus* specimens (181 and 182 mm TL) was recorded using a high-speed video camera (Sony RX100 IV). To achieve this, microalgal films were grown on glass Petri dishes for approximately three weeks. It is important to note that these microalgal films are not considered equivalent to turf algae (see [11] for a detailed discussion). Prior to experiments, fish were maintained for a week to ensure they were feeding reliably on a commercially available frozen food for herbivorous/detritivorous marine fishes. For the recording, a Petri dish covered with a microalgal film was suspended against the side glass of a 20 l aquarium containing a single *C. striatus* specimen. We recorded anterior views of feeding at 250 frames per second that allowed us to observe the mouth being pressed against the Petri dish. A total of 112 bites (fish 1, *n* = 52; fish 2, *n* = 60) were recorded. The video footage was then examined to determine the proportion of bites in which the RP margin (RPm; figure 1*b*) made full, partial or no contact with the glass.

Seven distinct, non-overlapping, bite marks (fish 1, *n* = 3; fish 2, *n* = 4) were photographed using a digital camera (Nikon D200, lens: Tamron SP 90 mm f/2.8 Macro) with a scale bar, and the relative area scraped by the top and bottom teeth calculated using Image J.

#### 2.3.2. Feeding observations in the wild

To examine the proportion of bites made using the chafing feeding mode, as described by Krone *et al*. [23], we used a focal animal approach [12] to assess feeding by *C. striatus.* Focal animals (*n *= 221) were observed feeding (mean of 46.1 ± 2.6 (SE) bites observed per fish) counting the number of bites made using the primary and chafing feeding modes. Care was taken to avoid observing the same individual twice. Observations were made in October 2016 between 09:00 and 17:00 on specimens which ranged in total length from 50 to 240 mm (estimated visually to the nearest 10 mm), in 1–5 m of water at 16 sites around Lizard Island in the northern GBR (electronic supplementary material, figure S1). The benthic covering of the feeding surfaces was predominantly short EAMs and/or crustose coralline algae.

## 3. Results

### 3.1. Anatomical observations

The RPs of the 12 *C. striatus* specimens were positioned on the anterior portion of the upper jaw with the apex pointing anteriorly and located immediately posterior to the premaxillary teeth (figure 1*b*). In all cases, the RP was soft to touch and was covered in soft papillae.

### 3.2. Scanning electron microscopy

Both of the RPs examined had a pronounced band of papillae (approximately 150 µm in height) along the anterior RPm (figure 1*c,d*; electronic supplementary material, figure S2a,b).

### 3.3. Histological observations

**Figure 2.**
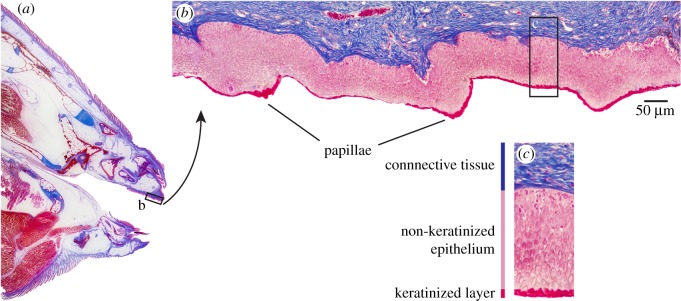
(*a*) Ayoub–Shklar stained cross section of the buccal region of the surgeonfish *Ctenochaetus striatus*. (*b*) Ayoub–Shklar stained cross section of the RP of *C. striatus*. Note the thin layer of keratinized cells (brilliant red). (*c*) The relative thickness of the keratinized cell layer compared to the non-keratinized epithelium.

The RP of *C. striatus* was covered with a stratified squamous epithelium that has columnar cells in the deeper layers. No acidic or neutral mucus-secreting cells were detected in the RP using the AB-PAS stain. However, the outermost layers of the epidermis in the RP stained brilliant red with the Ayoub–Shklar technique, indicating the presence of keratin. This stain revealed multiple cell layers of keratinocytes from immediately posterior to the teeth all the way to the velum at the posterior end of the oral cavity (figure 2; electronic supplementary material, figure S3a,b). This keratinized layer was on average 6.9 ± 1.7 (±s.e.) µm thick and was thickest (up to 21.1 µm thick) near the tips of the papillae. Taste buds were observed posterior to the RP, but not on the RP (electronic supplementary material, figure S3c).

### 3.4. Three-dimensional modelling and density estimates

**Figure 3.**
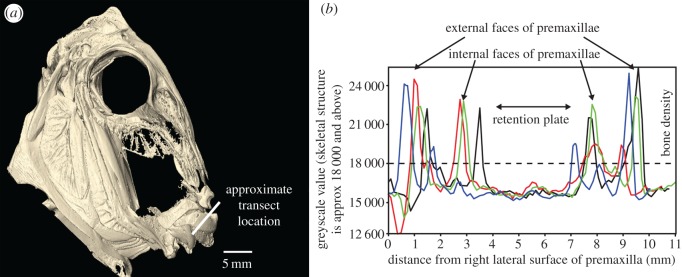
(*a*) A 3D model of the skull of *Ctenochaetus striatus*, generated using micro-computed tomography, showing where the transects were taken (electronic supplementary material, figure S5). (*b*) Four transects of greyscale data running from the right premaxilla towards the left premaxilla through the RP (the density of bone is conservatively estimated at 18 000) with densities across the RP matching that of surrounding soft tissues.

The 3D model of *C. striatus* revealed a lack of hard structures in the RP (figure 3; electronic supplementary material, figure S5), with densities (indicated by greyscale values) across the palate matching those of surrounding soft tissues (figure 3*b*).

### 3.5. Feeding observations in aquaria

**Figure 4.**
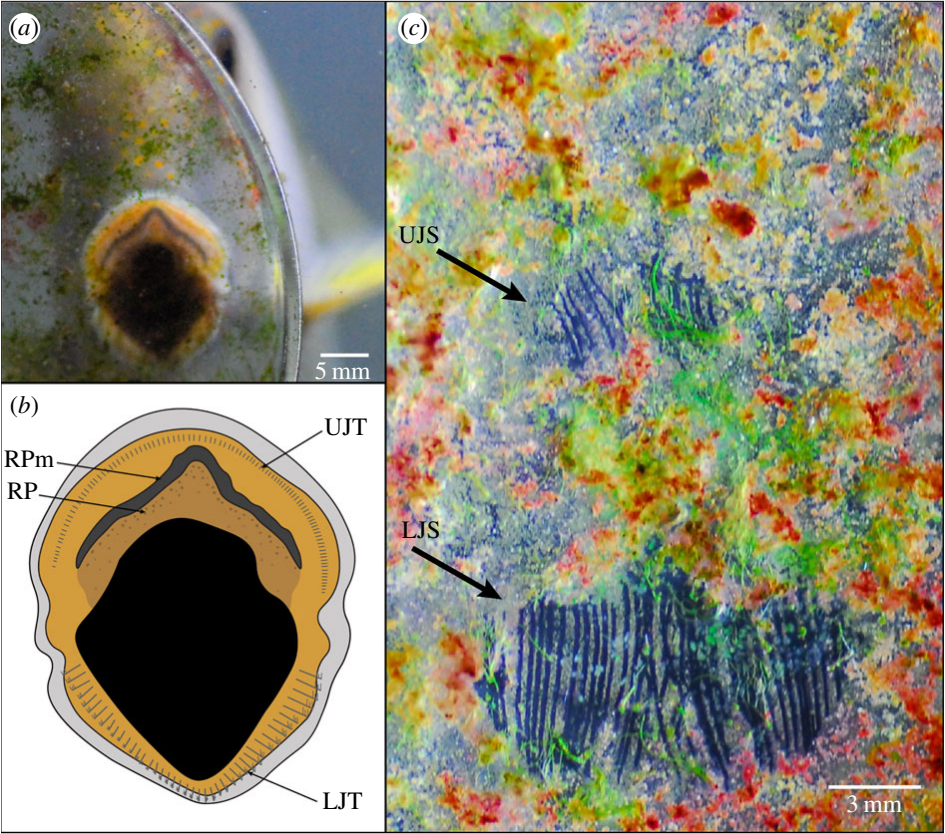
(*a*) *Ctenochaetus striatus* feeding on a microalgal film growing on a Petri dish. (*b*) Details of the mouth of *C. striatus* when in contact with the glass while feeding; LJT, lower jaw teeth; UJT, upper jaw teeth; RP, retention plate; RPm, retention plate margin. (*c*) Feeding scrapes from the teeth of *C. striatus* when removing the microalgal film; UJS, upper jaw scrape; LJS, lower jaw scrape. Note the relative size of the upper and lower jaw scrapes, the pointed upper margin of the lower jaw scrape mark and the lack of any dislodged material associated with the location of the RP structure.

Of the 112 bites recorded in slow motion, 99.1% resulted in the RPm (figures 1*b* and 4*a*,*b*) making contact with the glass, 87.5% were full contact, i.e. the RPm appeared to be firmly pressed against the glass (figure 4*a,b*), and 11.6% were partial contact. In only one instance did the RPm not appear to make contact with the glass. When contacting the glass Petri dish the RPm remained largely stationary and was not ‘rubbed’ against the glass.

There was little upper jaw movement during biting and examination of scrape marks indicated that the teeth of the lower jaw scraped 81.9 ± 0.1% (mean ± s.e.) of the total bite area. Upper jaw motion was minimal during biting (figure 4*c*) and was largely restricted to immediately before jaw closure. Numerous scrape marks showed no evidence of scraping by upper jaw teeth (electronic supplementary material, figure S4). Most importantly, there was no evidence that the microalgal film had been dislodged by the RP in any of the scrape marks examined.

### 3.6. Feeding observations in the wild

We did not observe use of the chafing feeding mode by *C. striatus* in any of the 10 204 bites observed in the wild at Lizard Island. All bites involved a quick closure of the lower jaws with minimal movement of the upper jaws following contact with the substratum.

## 4. Discussion

The RP in *Ctenochaetus striatus* is pronounced in comparison to those in other surgeonfishes [25] and it appears to serve an important function during feeding in this species. Based on four lines of evidence (anatomical examination, SEMs, histology and micro-CT scanning) we found no evidence that the RP was a rigid structure; however, we did find that the RP was lightly keratinized and covered in papillae. Furthermore, no evidence of the chafing feeding mode was observed in 10 204 bites of wild *C. striatus* on the GBR. Closer examination of feeding in *C. striatus* suggests that during a bite the lower jaw is responsible for removing approximately 80% of all ingested material. We also revealed that during these bites the RP touches the surface (fully or partially) in 99.1% of cases. We suggest the soft, lightly keratinized RP on the palate of *C. striatus* may play an important role in feeding, but is not used for bioerosion.

While the RP has previously been described as rigid [23], our evidence indicates that the RP of GBR specimens is composed of soft tissue covered by a thin veneer of keratinized cells. This keratinized layer on the RP is approximately half the thickness of the keratinized stratum corneum skin layer on human forearms (means of 6.9 versus 12.9 µm, respectively) [42]. Although such keratinization of palatal tissues is well known among other vertebrates [32–34], its extent and role in the palate of coral reef fishes have received little attention.

In fishes, a coat of mucus often lubricates and protects surfaces prone to abrasion or other mechanical lesions [43]. However, in terrestrial vertebrates keratinization is a common adaptation of the epidermis, especially where permeability is critical [44]; it is also found in palatal tissues [32–34]. Although rare, keratinization also occurs in the epithelial layers of a few fishes [45–48]. We did not detect mucus secretion in the RP of *C. striatus*. Instead, the keratinization observed in the epithelium suggests that the RP of *C. striatus* functions as a specialized surface tissue that is capable of withstanding mild abrasion. As the RP invariably contacts the substratum when feeding, such a protective surface is logical, especially considering the hard, abrasive nature of the reef matrix on which *C. striatus* predominantly feeds [8,49]. The relatively thin layer of keratinization further highlights the minimal movement that the RP makes when in contact with the substratum, i.e. the keratinized layer is likely to have a protective capacity when the RP is pushed against the substratum, but not if the RP is rubbed against it.

As *C. striatus* are highly selective feeders [50], it could be speculated that the RP may play some role in locating suitable feeding locations when in contact with the substratum. However, it appears that the RP does not provide taste information as taste buds were not located within the structure. In terms of the location of taste buds, our results align with those of Fishelson & Delarea [25] who found that they are located posterior to the RP, further back in the buccal cavity. Therefore, only once material enters the buccal cavity may these taste buds play a role in determining the quality of ingested material.

Our data suggest that the primary role of the RP is linked with the elongated teeth on the lower jaw of *C. striatus* during feeding. The major feeding mode in *C. striatus* involves contact of the mouth with the substratum and then a quick closure of the lower jaw. We found that this was the sole feeding mode in wild specimens, which is consistent with the observations of previous studies in the Indo-Pacific [19–21,49,51]. Furthermore, based on video examination and analysis of *C. striatus* scrape marks, we found that, on average, the lower jaw is responsible for 81.9% of the total bite. In nearly all cases (99.1%) the RPm makes complete or partial contact with the substratum during such bites, but it does not move. Based on the contribution of the lower jaw to feeding and the contact of the RPm with the substratum we infer that the RP acts primarily as a ‘dustpan’ in a ‘dustpan and brush’ feeding mechanism.

Previously, it was thought that two brushes (the elongated teeth on the lower and upper jaws of *C. striatus*) brushed material from the EAM together, with the lower jaw teeth playing the main role [21]. However, brushing material against a surface (the RP) rather than an opposing brush (the upper jaw teeth) may be more efficient in removing particulates from the EAM. This action is supported by the points on the upper margin of the scrapes left by the lower jaw teeth (figure 4*c*; electronic supplementary material, figure S4). These pointed scrapes suggest that particulate material dislodged by the teeth would be concentrated medially and funnelled into the mouth as the lower jaw teeth and the RP come together during the feeding process in a dustpan and brush action (see electronic supplementary material, figure S4). The upper jaw teeth merely provide an additional brushing action during the final stages of jaw closure.

Most fishes almost exclusively use hard structures for feeding [52]. However, our findings show that the soft tissue of the RP may play an important role in the feeding mechanism of *C. striatus*. Recent work has begun to highlight the importance of soft tissue structures in feeding. Indeed, Huertas & Bellwood [35] recently revealed the importance of the lips in the feeding ecology of the corallivorous tubelip wrasse, *Labropsis australis*. In this case, the grooved lips were responsible for forming a seal on the coral, facilitating the removal of coral mucus and tissue off the coral surface [35]. Specialized lip structures are also used by the suckermouth catfishes (Loricariidae) in freshwater systems. While many of these catfishes possess elongated teeth similar to those of *Ctenochaetus* [22,53,54], the catfishes feed quite differently, utilizing modified lip discs to adhere to the substratum while feeding [55,56]. The lips of suckermouth catfishes are covered in papillae and like the adhesive discs of Gobiesocidae [57] this morphology helps the fish ‘seal’ to uneven surfaces. The papillae on the RP of *C. striatus* may also help form a seal with the substratum, enhancing the funnelling of dislodged particulates into the mouth.

The similarity between the RP of *C. striatus* and the soft structures on the roof of the pharynx in some fishes, such as the palatal organ (PO) in Cypriniformes [27] and the pharyngeal pads in certain particulate-feeding fishes [58,59], is also noteworthy. Of these structures the PO has received considerable attention in the literature. The PO is a muscular pad located on the dorsal surface of the pharynx, opposing the branchial basket [27]. Generally, the PO is wider anteriorly and narrower posteriorly in a sub-rectangular or cordate shape, covered in papillae and taste buds [27,28,60,61]. The PO is largely used by Cypriniformes for tasting and sorting food items from inorganic material such as sediment [28,60]. The muscular, papillate nature of the PO is similar to the RP and may share a role in sorting material (see discussion below). However, the two structures differ in that the RP of *C. striatus* is located in the oral cavity, anterior to the vomer, i.e. in a more anterior position to the PO. The RP also lacks taste buds and is associated closely with the oral teeth, which are absent in the Cypriniformes [27]. While the RP and PO appear to be distinct structures, the utilization of morphologically similar soft tissue structures by phylogenetically disparate fishes is interesting.

The RP of *C. striatus* may not only aid in procuring particulates, but it could also assist in sorting sediment by type and particle size. A similar mechanism may exist in the pharyngeal valve of parrotfishes [17] and the PO of Cypriniformes [61]. On coral reefs, sediments are readily trapped within the EAM [62–64] from which *C. striatus* brush detrital and particulate material. *Ctenochaetus striatus* appear to be highly sensitive to variation in EAM sediment loads and particle size distributions [50,65]. This sensitivity is probably driven by the need to selectively feed on organically rich particulate material [50,65], while avoiding the ingestion of coarse, abrasive sediments that could damage their thin intestines [49]. Although the reef crest habitats where *C. striatus* predominantly feed are dominated by coarser sediments [62,64], the guts of *C. striatus* are generally filled with fine sediments and detrital material [14,66,67]. In a previous study, it was suggested that this would require selective feeding and sorting of ingested sediments [50]. Here we propose that a soft, flexible structure covered in papillae in the oral cavity could facilitate this selective sorting, ingestion and/or rejection of ‘lower quality’ sediments or particulates by retaining larger algal or sediment particles for subsequent ejection as previously observed in *C. striatus* [21,50,65].

While Krone *et al*. [23] and Schuhmacher *et al*. [24] suggest that the RP of *C. striatus* is used in bioerosion in the Red Sea, we found no evidence of this role in specimens examined from the GBR. During feeding, the RP did not dislodge microalgal films growing on glass. An observation that is supported by recent experimental work which demonstrated that *C. striatus* from the GBR are not capable of removing significant quantities of turf algae [11]. Furthermore, the chafing feeding mode which *C. striatu*s are reported to use to generate contact force with the substratum was not observed in the wild (it has only been observed on a handful of occasions in GBR *C. striatus* in aquaria; S.B.T., personal observation). Hence, the soft RP of *C. striatus* on the GBR is probably involved in the procurement of particulate material rather than bioerosion.

We aimed to determine the functional role of the RP and any chafing behaviour used during feeding by *C. striatus*. Our results suggest that this species uses the RP as a ‘dustpan’ which is closely linked to the opposing brushing action of the elongated teeth on the lower jaw. By concentrating and funnelling particulates into the mouth, the RP of *C. striatus* appears to facilitate the procurement of particulate material. The use of this soft structure in *C. striatus* for procurement of particulates further supports the suggestion of Clements *et al*. [17] that a closer examination of soft tissues in herbivorous/detritivorous coral reef fishes may yield new insights into their trophic ecology.

## Supplementary Material

Supplemental table and figures (Table S1, Figures S1-S4)

## Supplementary Material

Raw data (Tables S2-S5)

## Supplementary Material

Figure S5

## Data accessibility

The raw data from the study have been uploaded as electronic supplementary material (tables S2–S5).
