# Supplementary material for: A functional evaluation of feeding in the surgeonfish Ctenochaetus striatus: the role of soft tissues
Source: R Soc Open Sci. 2018 Jan 31;5(1):171111. doi: 10.1098/rsos.171111 (PMC5792902; doi:10.1098/rsos.171111)
Supplement: Supplemental table and figures (Table S1, Figures S1-S4) [file rsos171111supp1.docx]

**Electronic Supplementary Material**

Tebbett SB, Goatley CHR, Huertas V, Mihalitsis M, Bellwood DR. A functional evaluation of feeding in the surgeonfish *Ctenochaetus striatus*: the role of soft tissues. *R. Soc. open sci.*

**Table S1** The size range of the *Ctenochaetus striatus* specimens examined using each method.

| **Method** | **Size range (total length [mm])** | **Number of specimens** |
| --- | --- | --- |
| Anatomical observations | 145 - 235 | 12 |
| Scanning electron microscopy | 189 - 200 | 2 |
| Histology | 181 - 235 | 2 |
| 3D modelling | 170 | 1 |
| Filming in aquaria | 181 - 182 | 2 |
| Scrape marks on glass | 182 - 198 | 2 |


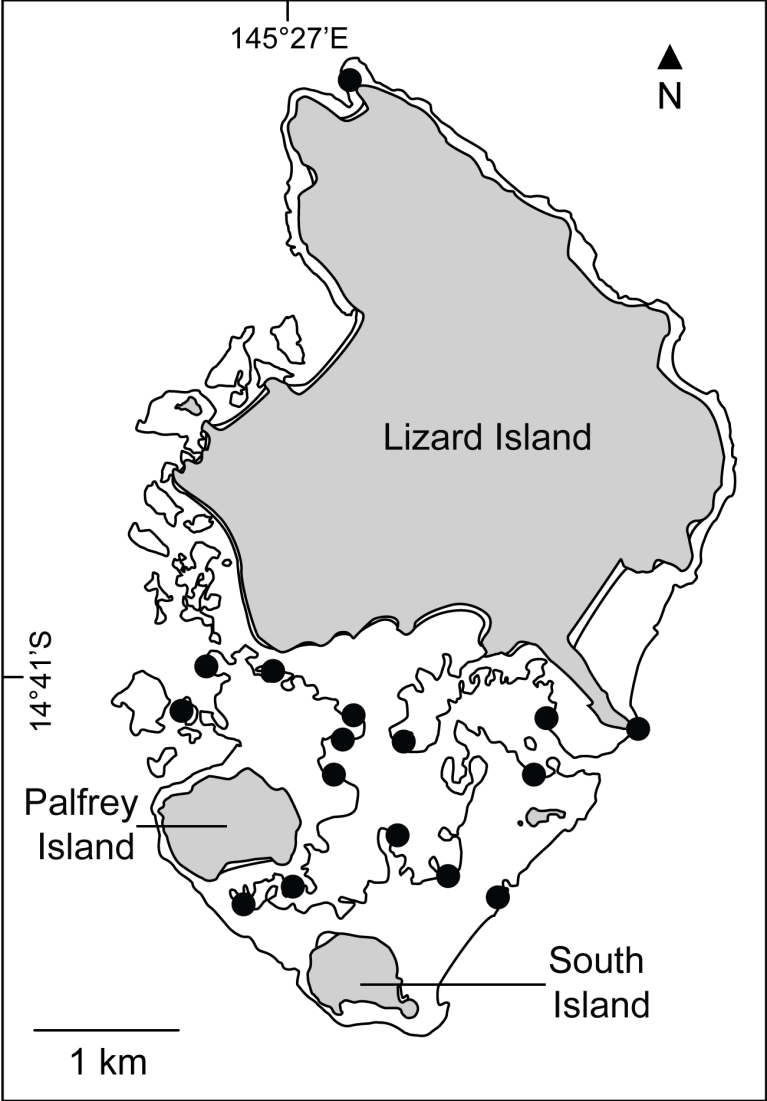


**Figure S1.** The 16 feeding observation study sites around Lizard Island


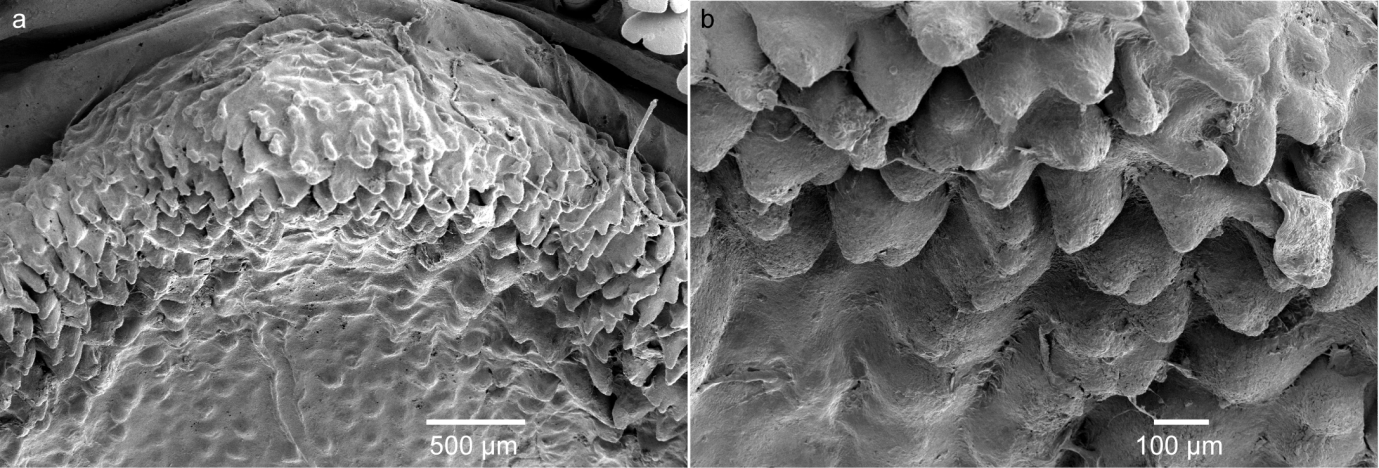


**Figure S2.** (a) scanning electron micrographs (SEM) of the retention plate of *Ctenochaetus striatus*, (b) close up SEM of papillae covered surface of the retention plate.


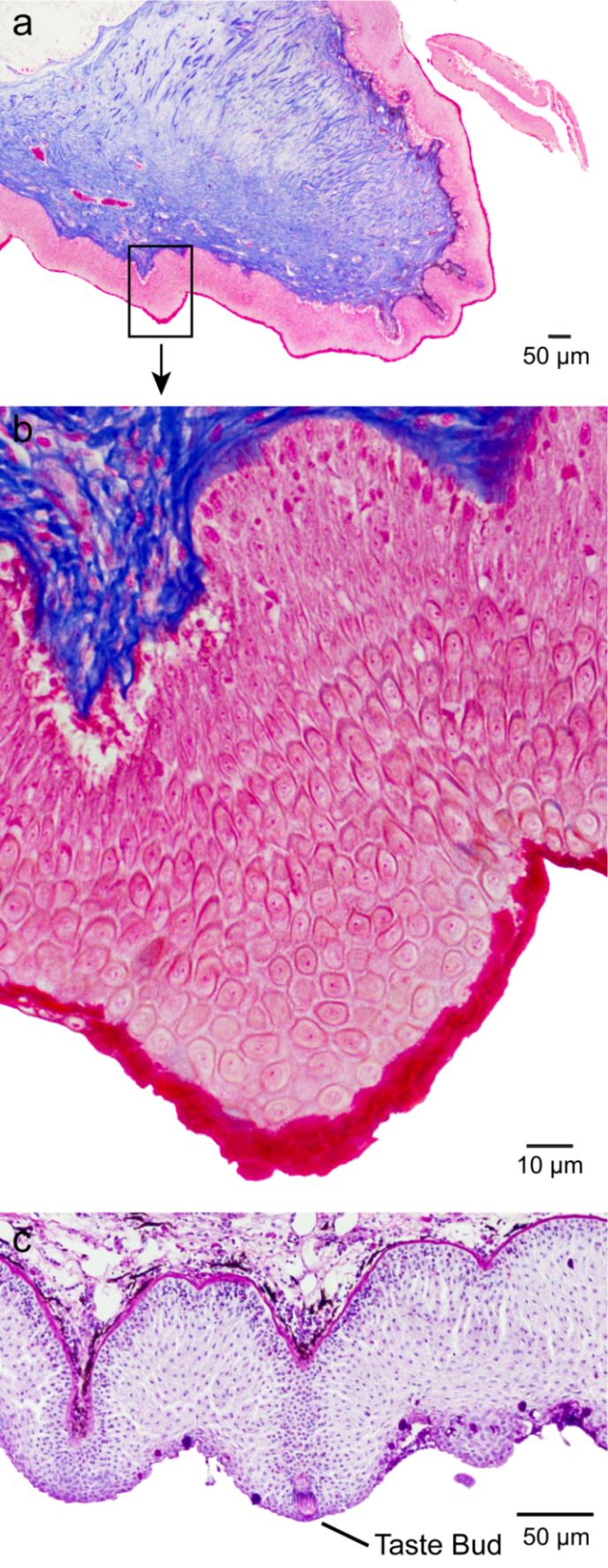


**Figure S3.** (a) Ayoub-Shklar stained cross section of the retention plate of *Ctenochaetus striatus*, (b) Ayoub-Shklar stained cross section of a papillae on the retention plate, (c) Alcian blue-PAS stained cross section showing a taste bud in the epithelium behind the retention plate.


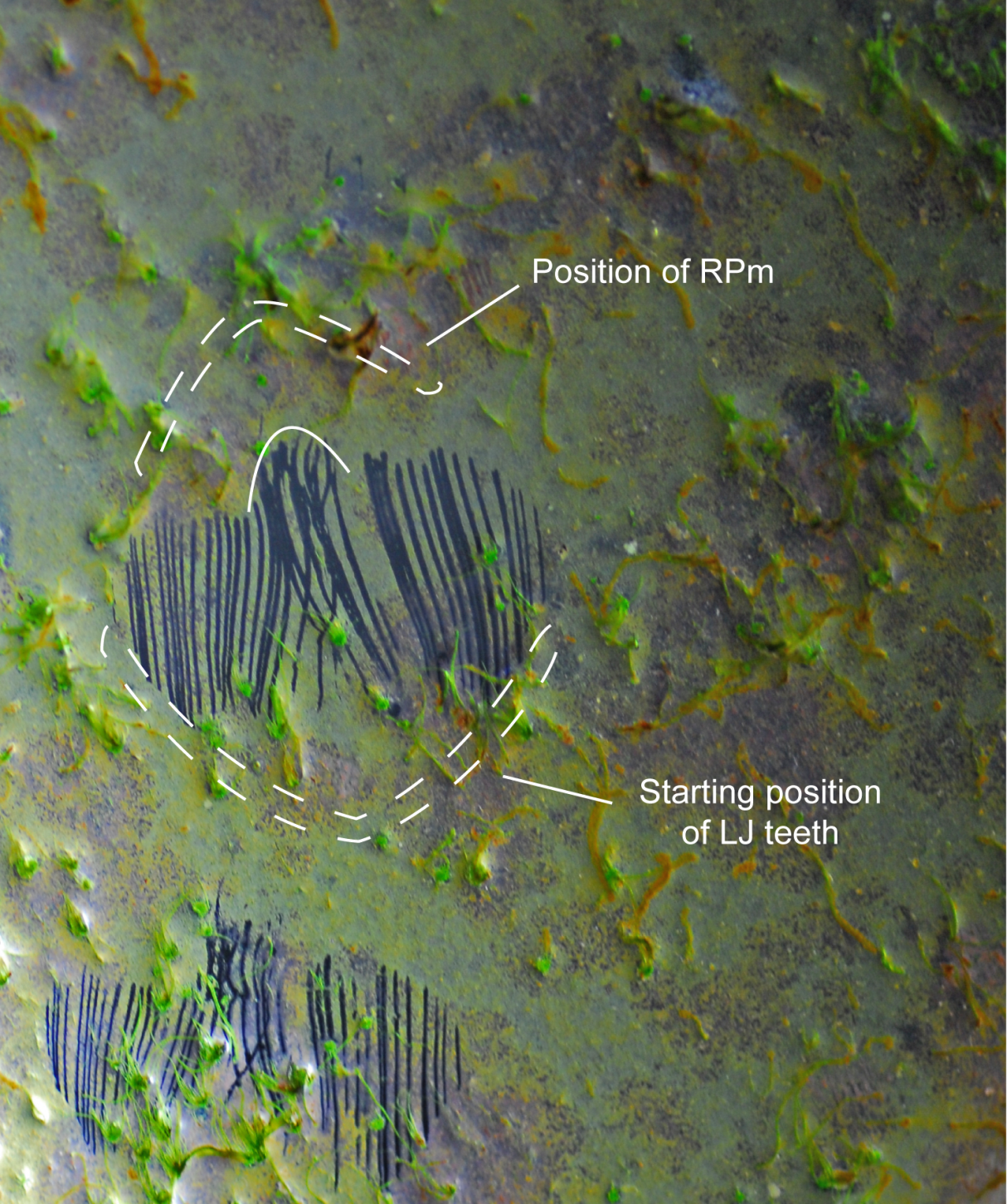


**Figure S4.** Two scrape marks left in a microalgal film on a glass petri dish, by the lower jaw teeth of the surgeonfish *Ctenochaetus striatus*. Note the middle teeth of the lower jaw (LJ) protrude into the position of the retention plate margin (RPm), leaving a distinct pointed section on the upper margin of the lower jaw scrape mark (indicated by the solid white line).
