# Supplementary material for: A functional evaluation of feeding in the surgeonfish Ctenochaetus striatus: the role of soft tissues
Source: R Soc Open Sci. 2018 Jan 31;5(1):171111. doi: 10.1098/rsos.171111 (PMC5792902; doi:10.1098/rsos.171111)
Supplement: Raw data (Tables S2-S5) [file rsos171111supp2.pdf]

Raw Data

Tebbett SB, Goatley CHR, Huertas V, Mihalitsis M, Bellwood DR. A functional evaluation of feeding in the surgeonfish *Ctenochaetus striatus*: the role of soft tissues. *R. Soc. open sci.*

Table S2 The thickness of the keratinised cell layer in the retention plate of *Ctenochaetus striatus.*

**Measurement number**	**Thickness (μm)**
1	5.276
2	4.976
3	21.142
4	4.047
5	0.811
6	6.709
7	4.575
8	5.709
9	7.331
10	8.827

Table S3 The number of bites in which the retention plate of *Ctenochaetus striatus* specimens made contact with a glass petri dish.

**Contact**	**Fish 1**	**Fish 2**
No	1	0
Partial	5	8
Full	46	52

Table S4 The proportion of the total area scraped by the lower jaws of *Ctenochaetus striatus* during a bite.

**Fish**	**Proportion**
1	0.826
1	0.809
1	0.867
2	0.773
2	0.821
2	0.793
2	0.842

Table S5 Feeding observations of *Ctenochaetus striatus* from around Lizard Island, Australia.

**Site**	**Fish Size (cm)**	**Primary Bites**	**Chafing Bites**
a	17	33	0
a	14	15	0
a	16	16	0
a	11	13	0
a	16	19	0
b	18	23	0
b	10	21	0
b	18	29	0
b	8	88	0
b	12	42	0
b	8	25	0
b	15	10	0
b	14	15	0
b	22	88	0
b	15	121	0
b	12	46	0
b	7	51	0
b	13	21	0
b	10	31	0
b	12	34	0
b	13	22	0
c	15	19	0
c	13	55	0
c	14	28	0
c	12	79	0
d	12	18	0
d	16	17	0
d	7	21	0
d	15	18	0
d	17	10	0
e	16	69	0
e	18	65	0
e	19	38	0
e	10	13	0
e	19	29	0
e	8	26	0
e	8	23	0
e	10	25	0
e	18	24	0
e	12	18	0
e	20	75	0

e	12	30	0
e	21	10	0
e	17	14	0
e	19	14	0
e	16	35	0
e	11	20	0
e	12	58	0
f	11	46	0
f	16	39	0
f	13	122	0
f	14	55	0
f	9	35	0
f	14	26	0
f	12	30	0
f	10	49	0
f	16	44	0
f	17	28	0
f	9	127	0
f	11	119	0
f	15	16	0
f	17	41	0
f	10	49	0
g	15	17	0
g	15	26	0
g	14	59	0
g	16	90	0
g	16	28	0
g	7	28	0
g	17	94	0
g	13	14	0
g	11	180	0
g	10	226	0
g	15	16	0
g	9	39	0
g	10	32	0
g	15	187	0
g	6	43	0
h	14	11	0
h	11	74	0
h	15	10	0
h	7	23	0
h	15	100	0
h	13	28	0
h	10	56	0

h	16	29	0
h	14	33	0
h	12	94	0
h	10	76	0
h	16	17	0
h	15	27	0
h	10	135	0
h	17	38	0
h	15	25	0
h	11	12	0
h	12	13	0
i	17	79	0
i	18	43	0
i	10	74	0
i	15	65	0
i	8	201	0
i	10	67	0
i	16	43	0
i	20	45	0
i	18	77	0
i	12	16	0
i	15	18	0
i	20	20	0
j	12	25	0
j	12	45	0
j	11	28	0
j	10	11	0
j	8	46	0
j	11	12	0
j	11	36	0
j	13	68	0
j	8	100	0
j	12	51	0
j	10	116	0
j	12	24	0
j	10	32	0
j	10	26	0
j	5	104	0
j	10	26	0
k	7	66	0
k	12	54	0
k	24	14	0
k	11	25	0
k	12	52	0

k	8	57	0
k	19	16	0
k	14	110	0
k	14	29	0
k	8	54	0
k	11	57	0
k	16	53	0
k	13	16	0
k	16	18	0
k	19	82	0
k	10	19	0
k	12	16	0
k	17	56	0
k	10	14	0
k	13	15	0
k	10	138	0
k	15	111	0
k	15	13	0
k	14	50	0
l	8	14	0
l	10	38	0
l	10	28	0
l	11	20	0
l	15	39	0
l	15	22	0
l	16	32	0
l	18	17	0
l	16	15	0
l	9	10	0
l	13	13	0
l	16	151	0
l	17	14	0
l	16	162	0
l	19	50	0
l	10	103	0
l	11	118	0
l	12	52	0
l	19	17	0
l	11	36	0
l	11	66	0
m	16	30	0
m	10	48	0
m	18	46	0
m	18	54	0

m	11	28	0
m	14	49	0
m	14	50	0
m	9	45	0
m	13	18	0
m	11	21	0
m	16	18	0
m	19	13	0
m	8	45	0
n	8	10	0
n	14	15	0
n	10	65	0
n	11	109	0
n	11	37	0
n	12	10	0
n	8	30	0
n	14	20	0
n	16	24	0
n	9	43	0
n	17	82	0
n	17	78	0
n	16	90	0
n	15	20	0
n	16	23	0
n	15	106	0
n	17	69	0
n	15	60	0
n	16	15	0
o	16	20	0
o	8	11	0
o	11	46	0
o	10	120	0
o	13	18	0
o	10	92	0
o	11	104	0
o	16	65	0
o	13	81	0
o	8	37	0
o	8	15	0
p	16	15	0
p	9	16	0
p	6	48	0
p	15	37	0
p	15	20	0

p	12	29	0
p	11	41	0
p	15	15	0
p	15	33	0
